# Genetic Population Structure of *Macridiscus multifarius* (Mollusca: Bivalvia) on the Basis of Mitochondrial Markers: Strong Population Structure in a Species with a Short Planktonic Larval Stage

**DOI:** 10.1371/journal.pone.0146260

**Published:** 2015-12-31

**Authors:** Ying Ying Ye, Chang Wen Wu, Ji Ji Li

**Affiliations:** 1 National Engineering Research Center of Maricultural Facilities of China, College of Marine Science and Technology, Zhejiang Ocean University, Zhoushan, China; 2 Department of Aquatic Science, Faculty of Science, Burapha University, Chon Buri, Thailand; 3 Università degli studi di Napoli “Federico II”, Portici, Naples, Italy; University of California Santa Cruz, UNITED STATES

## Abstract

The clam *Macridiscus multifarius* with a planktonic larval stage of about 10 days is an ecologically and economically important species in the coastal regions of China. In this study, 3 mt-DNA markers (COI, 12S rRNA, and ND1) were used to investigate the population structure and demography of wild *M*. *multifarius* populations in 3 coastal localities of the East China Sea (ZS and ZP populations) and Beibu Gulf in the South China Sea (BH population). Sequences of 685 bp in COI, 350 bp in 12S rRNA, and 496 bp in ND1 were determined. High level and significant *F*_*ST*_ values were obtained among the different localities on the basis of either COI (*F*_*ST*_ = 0.100–0.444, *p <* 0.05) or 12S rRNA (*F*_*ST*_ = 0.199–0.742, *p <* 0.05) gene, indicating a high degree of genetic differentiation among the populations. *F*_*ST*_ values were significant but weak for the ND1 gene because it is highly conservative. The median-joining network suggested an obvious genetic differentiation between ZS and BH populations, and the finding is consistent with the results of our demographic analyses using the unweighted pair group method with arithmetic mean. Our study unraveled the extant population genetic structure of *M*. *multifarius* and explained the strong population structure of a species with a short planktonic larval stage species; this information could be useful for fishery management measures, including artificial breeding and conservation.

## Introduction

The life history of most marine organisms includes a planktonic stage during which larvae disperse to distances that range from several meters to hundreds of kilometers from their location of release [1]. In general, species with a long planktonic larval stage are capable of moving great distances and dispersing widely via ocean currents. The population genetic theory predicts that a longer planktonic larval stage would result in increased gene flow, and consequently, decreased levels of population differentiation. For example, a solitary coral species with a brief planktonic larval stage showed a stronger population genetic structure than another coral species with a longer larval stage [2]. Shanks et al. [3] found that the mollusc *Haliotis rubra*, with a 6-day pelagic larval phase, has a dispersal distance of less than 15 km, that *Ensis directus*, with a 16-day pelagic larval phase, can disperse to about 111 km, and that *Perna perna*, with a 15 to 20-day pelagic larval phase, can disperse to about 235 km. Shanks et al. observed that propagules that spent in the water column dispersed further [3]. However, Galarza et al. [4] showed that the pelagic larval phase often fails to achieve complete dispersal potential. Thus, the relationship between the pelagic larval phase and gene flow in planktonic developers may be quite complex and needs to be studied further [4].

The clam *Macridiscus multifarius* L. F. Kong, Matsukuma & Lutaenko, 2012 [5], also called the sandy clam, belongs to the family Veneridae (Mollusca; Bivalvia; Veneridae; Macridiscus) and is known to be native to the coastal waters of the Western Pacific region, which extends from the coast of Japan to Northwestern Australia [6]. In China, *M*. *multifarius* is found along the coast, and it has a shell length of 30–40 mm. It has a brief pelagic phase of about 10 days at 24–29°C and 20–25 ppt of salinity. Spawning of *M*. *multifarius* occurs in summer and peaks in July and August [6]. The adults are benthic and relatively immobile, so patterns of larval dispersal and recruitment help in understanding the population connectivity of *M*. *multifarius*. This clam is an important and commercially exploited species; however, *M*. *multifarius* populations have declined largely because of over-exploitation and habitat destruction [6]. A previous study showed that the shell length of most *M*. *multifarius* specimens collected from Nanji Island, Zhejiang Province, was less than 15 mm [6]. The decline in *M*. *multifarius* populations has caused the price to increase, for example, in Zhoushan City, Zhejiang Province, the price has increased from ¥20 to more than ¥100 per kilogram in a few years [7]. Therefore, there is a great need to conserve and manage *M*. *multifarius* populations.

Enhancement practices that involve the release of *M*. *multifarius* juveniles into natural coastal areas were initiated by the Zhejiang Marine Aquaculture Research Institute in 2011 (about 6,156,000 individuals) and 2012 (about 2,155,000 individuals). *M*. *multifarius* is also bred artificially; however, very little is known about the population dynamics. Management and conservation efforts could be improved by examining how environmental factors influence population connectivity and patterns of population genetic structure [8]. Population genetic structure reflects the evolutionary history as well as the evolutionary potential of a species. Lack of information on the population genetic structure may lead to over-exploitation and subsequent resource collapse. The population genetic structure of marine organisms has been partially explained by biophysical factors such as the biology, ecology, and behavior of the species as well as hydrographical barriers to dispersal [9, 10].

Genetic markers are useful tools for measuring genetic variation and gene flow among populations [11]. In the present study, we used the gene sequences of partial mitochondrial cytochrome c oxidase subunit I (COI), ribosomal 12S subunit (12S rRNA), and dehydrogenase subunit 1 (ND1) to assess the genetic diversity and population structure of natural *M*. *multifarius* populations in China. The objective of this study was to test the influence of planktonic larval stage duration on the genetic structure of *M*. *multifarius*. Our approach could add in the design of spatial fishery management and conservation strategies for species that inhabit the East China Sea and South China Sea.

## Materials and Methods

### Study area and sampling

**Fig 1:**
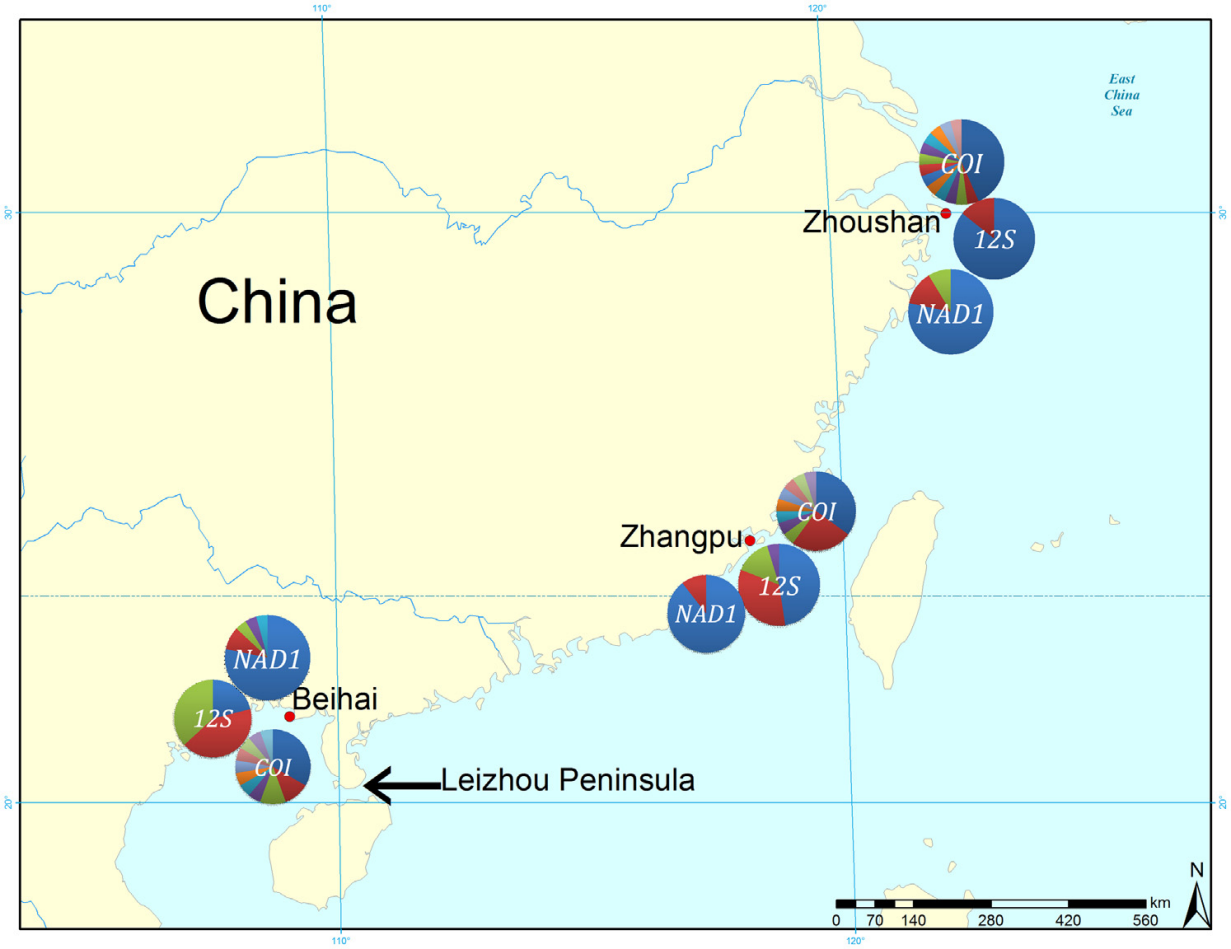
A map illustrating the three sample sites of *M*. *multifarius* populations in the East China Sea (Zhoushan [ZS] and Zhangpu [ZP]) and Beibu Gulf (Beihai [BH]). Different haplotypes for each gene have been separated by color, with the two most common haplotypes (H1 and H2) shown in blue and red, respectively.

All experiments and animal sampling were reviewed and approved by the State Oceanic Administration of China and the Ethics Committee of Zhejiang Ocean University and performed according to national laws and regulations. Wild adult specimens of *M*. *multifarius* were collected from 3 coastal localities in the East China Sea (Zhoushan [ZS], 29°35′ N, 122°25′ E, Zhejiang Province and Zhangpu [ZP] 23°32′ N, 117°58′ E, Fujian Province) and Beibu Gulf (Beihai [BH], 20°54′ N, 109°47′ E, Guangxi Province). These sites do not belong to a national park or a protected sea area or a relevant regulatory body concerned with wildlife protection or a private owner. We confirmed that the field studies did not involve endangered or protected species. Geographic locations and sample sizes of all the examined populations are provided in Fig 1 and Table 1. All the samples were collected in October 2014. Tissues from the adductor muscle were dissected from fresh specimens, preserved in 95% ethanol, and frozen at -20°C until DNA extraction.

**Table 1:** Collection Sites, Sample Sizes (No.), and Summary Statistics of Genetic Variability for *Macridiscus multifarius* (COI, 12S rRNA, and NDI).

Sequence	Population	No.	Haplotype															
			H1	H2	H3	H4	H5	H6	H7	H8	H9	H10	H11	H12	H13	H14	H15	H16
	Zhoushan	23	10	1	1	1	1	1	1	1	1	1	1	1	1	1		
COI	Zhangpu	20	7										5		1		1	1
	Beihai	18											6		2			
	Zhoushan	21	18	3														
12S	Zhangpu	21	10	7	3	1												
	Beihai	19					4	8	7									
	Zhoushan	23	18	3	2													
ND1	Zhangpu	19	17	2														
	Beihai	23	18	2	1	1	1											

Sequence	Population	No.	Haplotype													No. of Haplotypes	Haplotype diversity (h±SD)	Nucleotide diversity (π±SD)
			H17	H18	H19	H20	H21	H22	H23	H24	H25	H26	H27	H28	H29			
	Zhoushan	23														14	0.822 ± 0.083	0.004 ± 0.001
COI	Zhangpu	20	1	1	1	1	1									10	0.837 ± 0.065	0.006 ± 0.001
	Beihai	18		2				1	1	1	1	1	1	1	1	11	0.889 ± 0.064	0.004 ± 0.001
	Zhoushan	21														2	0.257 ± 0.110	0.001 ± 0.000
12S	Zhangpu	21														4	0.671 ± 0.066	0.003 ± 0.000
	Beihai	19														3	0.678 ± 0.051	0.002 ± 0.000
	Zhoushan	23														3	0.379 ± 0.117	0.001 ± 0.000
ND1	Zhangpu	19														2	0.199 ± 0.112	0.001 ± 0.000
	Beihai	23														5	0.391 ± 0.125	0.003 ± 0.001

### DNA extraction and PCR amplification

Total genomic DNA was extracted from the adductor muscle of *M*. *multifarius* by using the salt-extraction procedure [12] with slight modifications, and the tissues were pretreated with Proteinase K. The extracted DNA was stored in TE buffer at 4°C.

For all markers, PCR was performed using the Taq Master Mix (ComWin Biotech Co., Ltd., Beijing, China). The PCR primer sequences have been listed in Table 2. Amplification was performed using the Applied Biosystems Veriti 96-Well Thermal Cycler (Applied Biosystems, Inc., Foster City, CA, USA). Cycling conditions for all assays were as follows: initial denaturation at 94°C for 2 min; 35 cycles at 94°C for 30 s (denaturation), 50–54°C (Table 2) for 30 s (annealing), and 72°C for 45 s (elongation); and final elongation at 72°C for 7 min. All PCR products were checked for the presence of products of the correct size on 1% agarose gels (1× TBE) pre-stained with SYBR Safe dye (Invitrogen Corp., Carlsbad, CA, USA). DNA sequencing was performed by BGI Tech Solutions Co., Ltd. (Shanghai, China) with both forward and reverse primers. All the obtained sequences were deposited in GenBank under accession numbers KP699649–KP699689.

**Table 2:** Primers Used for the Amplification and Sequencing of COI, 12S rRNA, and NDI Sequences in *Macridiscus multifarius*.

DNA region	Primer and sequence (5’–3’)	Tm (°C)	Amplicon size (bp)	Reference
COI	COIL 1490: GGTCAACAAATCATAAAGATATTGG; COIH 2198: TAAACTTCAGGGTGACCAAAAAATCA	51	685	[13]
12S rRNA	G12SF2: TTGGCGRTTAAWTCGAT; G12SR6: TTACYATGTTACRACTTA	50	350	[14]
ND1	Gnad1F: CAWGGCCCWAATAARGT; Gnad1R4: GCCACYAAYTCWGACTC	54	496	[14]

### Data analysis

For all sequence analyses (COI, 12S rRNA, and ND1), genetic similarities were evaluated using BLAST (http://www.ncbi.nlm.nih.gov/BLAST) to identify *M*. *multifarius* sequences. Then, the sequences were aligned using Clustal W [15], and individual consensus sequences were retrieved using both alignment and manual checks. The aligned DNA sequences were imported into MEGA version 5.0 [16] for sequence comparisons and variation analysis. Standard genetic diversity indices, such as the number of haplotypes, polymorphic sites, haplotype diversity (*h*), and nucleotide diversity (*p*), were calculated with DnaSP 4.0 [17]. Tajima’s D [18] and Fu’s Fs [19] neutrality tests were performed using Arlequin version 3.11 [20] and 10,000 random permutations to infer population expansion events and check for deviations from a strictly neutral model of evolution.

Phylogenetic trees were constructed using the unweighted pair group method with arithmetic mean (UPGMA) [21] based on a matrix of the Kimura two-parameters (K2P) distance method [22] in MEGA 5.0 [16]. Statistical robustness in the nodes of the resulting tree was determined by 1000 bootstrap replicates [23]. To depict phylogenetic and geographical relationships of the haplotypic sequences, 3 haplotype networks of COI, 12S rRNA, and ND1 were created using the median-joining method in Network 4.6 software [24]. Population structures based on COI, 12S rRNA, and ND1 genes were investigated using analysis of molecular variance (AMOVA) [25] in Arlequin version 3.11 [20]. Genetic differentiation, genetic distance, and migration rate among the populations were estimated by calculating the F statistic (*F*_*ST*_) between the populations and testing their significance with 1000 permutations.

## Results

### Genetic variation

Sequences of the 685-bp COI gene were determined in 61 specimens, and 33 polymorphic sites and 29 haplotypes were detected. Twenty-five haplotypes were found in only one population, 2 (H1 and H18) were found to be shared by two populations, and 2 (H11 and H13) were found to be shared by all three populations (Table 3). A 350-bp fragment of 12S rRNA gene was sequenced from 61 samples, and 4 polymorphic sites and 7 haplotypes were detected. Two haplotypes (H1 and H2) were found to be shared by ZS and ZP populations; the rest were population specific haplotypes (Table 4). In addition, 496 bp of the ND1 gene was sequenced using 65 specimens, and 5 polymorphic sites and 6 haplotypes were detected. H1 and H2 were shared among all 3 populations; H3 was observed in ZS and BH populations; H4 and H5 were specific for the BH population (Table 5). Haplotype diversity (*h*) and nucleotide diversity (*π*) based on COI, 12S rRNA, and ND1 genes for the 3 populations are listed in Table 1.

**Table 3:** Variable Position of 29 Haplotypes of COI in *Macridiscus multifarius*.

Haplotype	Nucleotide position beginning from 5´ end																	GenBank accession number
	42	93	141	171	201	210	237	255	265	276	279	298	318	324	354	375	387	
H1	A	T	C	C	T	G	C	T	T	G	A	C	T	T	T	G	A	KP699649
H2	*	*	*	*	*	*	*	*	*	*	*	T	*	*	*	*	*	KP699650
H3	*	*	*	*	*	*	*	*	*	*	G	*	*	*	*	*	*	KP699651
H4	*	*	*	*	*	*	A	*	*	*	*	*	*	*	*	*	*	KP699652
H5	*	*	*	*	*	*	*	*	*	*	*	*	*	*	*	*	*	KP699653
H6	*	*	*	*	*	*	*	A	*	*	*	T	*	*	*	*	*	KP699654
H7	*	*	*	*	*	*	*	*	*	*	*	*	C	*	*	*	*	KP699655
H8	*	*	*	*	*	*	*	*	*	*	*	*	*	*	*	*	*	KP699656
H9	*	*	*	*	*	*	*	*	*	*	*	T	C	*	*	*	*	KP699657
H10	*	*	*	*	*	*	*	*	*	*	*	*	*	*	*	*	*	KP699658
H11	*	*	*	*	*	*	*	A	*	*	*	T	*	*	*	*	*	KP699659
H12	*	*	*	*	*	*	*	*	*	*	*	*	*	*	*	*	*	KP699660
H13	*	*	*	*	*	*	*	*	*	*	*	*	*	*	*	*	G	KP699661
H14	*	*	*	*	C	*	*	A	*	*	*	T	*	*	*	*	*	KP699662
H15	*	*	*	*	*	*	*	A	*	*	*	T	*	*	C	*	*	KP699663
H16	*	*	*	*	*	*	*	A	C	*	*	T	*	*	*	*	*	KP699664
H17	G	*	*	*	*	*	*	A	*	*	*	T	*	*	*	*	*	KP699665
H18	*	C	*	*	*	*	*	A	*	*	*	T	*	*	*	*	*	KP699666
H19	*	*	*	*	*	A	*	*	*	*	*	*	C	*	*	*	*	KP699667
H20	*	*	*	*	*	*	*	A	*	*	*	T	*	*	*	*	*	KP699668
H21	*	*	*	*	*	*	*	*	*	*	*	*	*	*	*	A	G	KP699669
H22	*	*	*	*	*	*	*	*	*	A	*	*	*	*	*	*	*	KP699670
H23	*	*	*	*	*	*	*	A	*	A	*	T	*	*	*	*	*	KP699671
H24	*	*	*	*	*	*	*	A	*	*	*	T	*	*	*	*	*	KP699672
H25	*	*	*	*	*	*	*	A	*	*	*	T	*	A	*	*	*	KP699673
H26	*	*	G	T	*	*	*	A	*	*	*	T	*	*	*	*	*	KP699674
H27	*	*	*	*	*	*	T	A	*	*	*	T	*	*	*	*	*	KP699675
H28	*	*	*	*	*	*	*	A	*	*	*	T	*	*	*	*	*	KP699676
H29	*	*	*	*	*	*	*	A	*	*	*	T	*	*	*	*	*	KP699677

Haplotype	Nucleotide position beginning from 5´ end																GenBank accession number
	417	432	438	459	468	474	492	501	550	567	570	576	583	591	624	669	
H1	A	T	C	T	T	G	A	C	T	T	C	T	T	G	T	G	KP699649
H2	*	C	*	*	*	*	*	*	*	*	*	*	*	*	*	*	KP699650
H3	*	*	*	*	C	*	*	*	*	*	*	*	*	*	*	*	KP699651
H4	*	*	*	*	*	*	*	*	*	*	*	*	*	*	*	*	KP699652
H5	*	*	*	*	*	*	*	*	*	*	T	*	*	*	*	*	KP699653
H6	G	*	*	*	*	*	*	*	*	C	*	*	C	A	*	A	KP699654
H7	*	*	*	*	*	*	*	*	*	*	*	*	*	*	C	*	KP699655
H8	*	*	*	*	*	*	*	*	*	*	*	*	*	*	C	*	KP699656
H9	*	*	*	*	*	*	*	*	*	*	*	*	*	*	C	*	KP699657
H10	*	C	*	*	*	*	G	*	*	*	*	*	*	*	*	*	KP699658
H11	G	*	*	*	*	*	*	*	*	C	*	*	*	*	*	A	KP699659
H12	*	C	*	*	*	*	*	*	*	*	*	*	*	*	*	*	KP699660
H13	*	*	*	*	*	*	*	*	*	*	*	*	*	*	*	*	KP699661
H14	G	*	*	*	*	*	*	*	*	C	*	*	*	*	*	A	KP699662
H15	G	*	*	*	*	*	*	*	C	C	*	*	C	A	*	A	KP699663
H16	G	*	*	C	*	A	*	*	*	C	*	*	*	*	*	A	KP699664
H17	G	*	T	*	*	*	*	*	*	C	*	*	*	*	*	A	KP699665
H18	G	*	*	*	*	*	*	*	*	C	*	*	*	*	*	A	KP699666
H19	*	*	*	*	*	*	*	*	*	*	*	*	*	*	C	*	KP699667
H20	G	*	*	*	*	*	*	T	*	C	*	*	*	*	*	A	KP699668
H21	*	*	*	*	*	*	*	*	*	*	*	*	*	*	*	*	KP699669
H22	*	*	*	*	*	*	*	*	*	*	*	*	*	*	*	*	KP699670
H23	G	*	*	*	*	*	*	*	*	C	*	*	*	*	*	A	KP699671
H24	G	*	*	*	*	*	*	*	*	C	*	C	*	*	*	A	KP699672
H25	G	*	*	*	*	*	*	*	*	C	*	*	*	*	*	A	KP699673
H26	G	*	*	*	*	*	*	*	*	C	*	*	*	*	*	A	KP699674
H27	G	*	*	*	*	*	*	*	*	C	*	C	*	*	*	A	KP699675
H28	G	*	*	*	*	*	*	*	*	C	T	*	*	*	*	A	KP699676
H29	G	*	*	*	*	*	*	*	*	*	*	*	*	*	*	A	KP699677

*means that the nucleotide is the same as the nucleotide of H1.

**Table 4:** Variable Positions of 7 Haplotypes of 12S rRNA *in Macridiscus multifarius*.

Haplotype	Nucleotide position beginning from 5´ end				GenBank accession number
	118	207	230	345	
H1	T	G	A	G	KP699678
H2	*	*	*	*	KP699679
H3	*	*	G	*	KP699680
H4	C	*	*	*	KP699681
H5	*	G	*	A	KP699682
H6	*	*	G	A	KP699683
H7	*	*	*	A	KP699684

*means that the nucleotide is the same as the nucleotide of H1.

**Table 5:** Variable Position of 5 Haplotypes of ND1 *in Macridiscus multifarius*.

Haplotype	Nucleotide position beginning from 5´ end						GenBank accession number
	110	191	227	260	308	437	
H1	C	T	A	T	A	A	KP699685
H2	T	*	*	*	*	G	KP699686
H3	T	*	*	*	*	*	KP699687
H4	T	*	C	C	G	G	KP699688
H5	T	C	C	C	G	G	KP699689

*means that the nucleotide is the same as the nucleotide of H1.

Not all of Tajima’s D results for the 3 genes were significant (*p* > 0.05; Table 6), indicating balanced selection and/or a decrease in population size [18]. Most of Fu’s F_S_ results for the 3 genes were not significant (*p* > 0.05; Table 6), except F_S_ results for the COI gene in the ZS (*p* < 0.01) and BH (*p* < 0.05) populations; this implies that *M*. *multifarius* may undergo population selection or expansion events.

**Table 6:** Neutrality Test Results for COI, 12S rRNA, and NDI Data Obtained for *Macridiscus multifarius*.

Test	COI			12S rRNA			NDI		
	ZS	ZP	BH	ZS	ZP	BH	ZS	ZP	BH
Tajima’s D	-1.476	-1.030	-1.050	-0.133	0.133	1.225	0.210	-0.730	-0.724
*p*	0.051	0.156	0.170	0.343	0.607	0.860	0.654	0.250	0.271
Fu’s Fs	-7.924	-1.366	-4.580	0.341	-0.261	0.847	0.217	0.960	-0.381
*p*	0.000**	0.265	0.007*	0.346	0.382	0.654	0.461	0.521	0.407

* Significant differentiation (*p* < 0.05).

** Highly significant differentiation (*p* < 0.01).

### Haplotype network analysis

**Fig 2:**
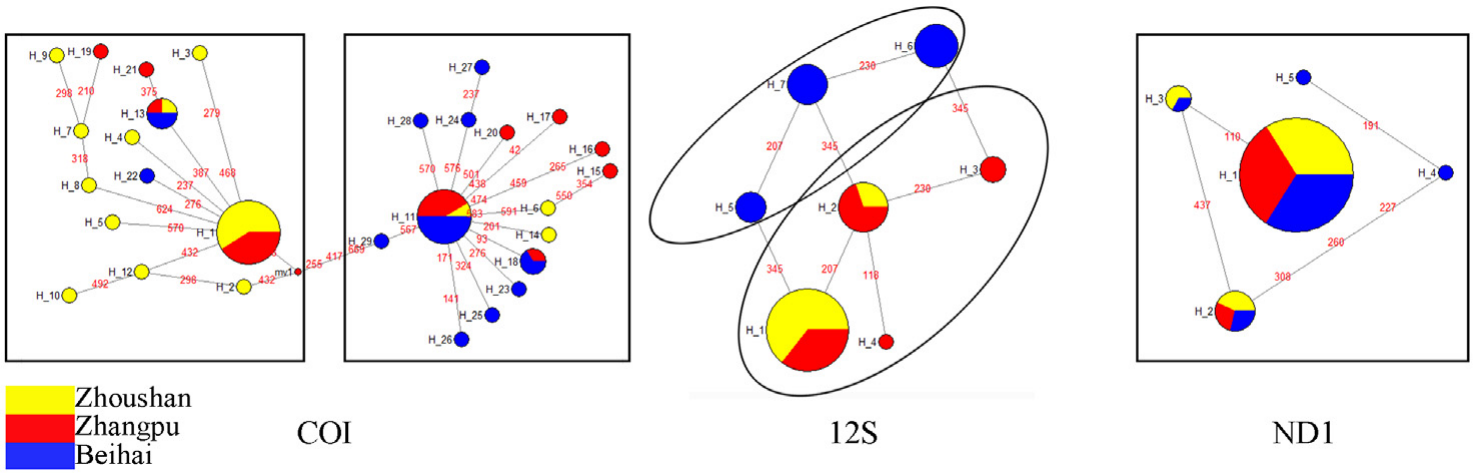
Median-joining network for COI, 12S rRNA, and NDI haplotypes of *M*. *multifarius*. On the connecting lines, red numbers present the variable sites between each haplotype pair. Different colors represent the 3 populations in the network.

The median joining network (Fig 2) illustrates the polymorphic sites, including the number and frequency of the haplotypes for COI, 12S rRNA, and ND1 sequences. The COI network was radial-like with a high number of unique haplotypes closely related to 2 central haplotypes (H1 and H11). Dominant haplotypes H1 and H11 accounted for 27.87% (17/61) and 19.67% (12/61), respectively, of all 61 specimens; this also suggested that H1 and H11 are ancestral haplotypes. The ZS population was separated from the BH population. The 12S rRNA network can also be divided into 2 parts: the left rectangle consists of blue haplotypes, while the right circle consists of yellow and red haplotypes; this suggested that the BH population was separated from ZS and ZP populations. Because of fewer haplotypes, the ND1 network shows a single line, with the dominant haplotype H1 accounting for 81.54% (53/65) of all specimens.

### Population genetic structure

Significant *F*_*ST*_ values were observed in all pairwise comparisons between populations for the COI gene (*p* < 0.05; Table 7). Pairwise *F*_*ST*_ values ranged from 0.100 to 0.444, indicating great genetic differentiation between ZS and BH populations (*F*_*ST*_ = 0.444); *N*_*m*_ was 0.627, indicating very low gene flow between these 2 localities. The other 2 pairwise *N*_*m*_ values (ZS and ZP populations and ZP and BH populations) were > 1, indicating an extensive genes flow among the populations. The same pattern was found using 16S gene data: pairwise *F*_*ST*_ ranged from 0.200 to 0.742 and pairwise *N*_*m*_ ranged from 0.174 to 2.012. The maximum *F*_*ST*_ value was also found between ZS and BH populations, while the *N*_*m*_ value was < 1. For ND1 gene data, the genetic structure was weak but significant and no differentiation was detected among the populations (*F*_*ST*_ < 0.05).

**Table 7:** *F*_ST_ Value and Gene Flow among the 3 Populations of *Macridiscus multifarius*.

Gene	COI			12S rRNA			NDI		
Population	ZS	ZP	BH	ZS	ZP	BH	ZS	ZP	BH
ZS	--	3.263	0.627	--	2.012	0.174	--	∞	∞
ZP	0.133*	--	4.503	0.199*	--	0.394	-0.022*	--	942.896
BH	0.444*	0.100*	--	0.742*	0.560*	--	-0.015*	0.001*	--

^1)^ Data above the diagonal are *N*_*m*_ values, and data below the diagonal are *F*_ST_ values.

* indicates the significance level of *F*_ST_ value at *p* < 0.05.

AMOVA for COI, 12S rRNA, and ND1 genes on the basis of haplotype frequencies revealed that 75.56%, 41.98%, and 101.1%, respectively, of the genetic variation occurred within the populations, whereas 24.44%, 58.02%, and -1.1%, respectively, of the genetic variation occurred among the populations (Table 8). AMOVA results for overall population genetic structure within and among the populations were highly significant (*p* < 0.001), except for ND1 gene results.

**Table 8:** Analysis of Molecular Variance Performed Using *Macridiscus multifarius* Populations.

Gene	Source of variation	df	Variance component	Percentage (%)	*p*
	Among populations	2	0.535	24.44	< 0.001
COI	Within populations	58	1.655	75.56	< 0.001
	Total	60	2.191		
	Among populations	2	0.456	58.02	< 0.001
12S rRNA	Within populations	58	0.330	41.98	< 0.001
	Total	60	0.785		
	Among populations	2	-0.004	-1.10	> 0.001
ND1	Within populations	62	0.383	101.10	> 0.001
	Total	64	0.379		

**Fig 3:**
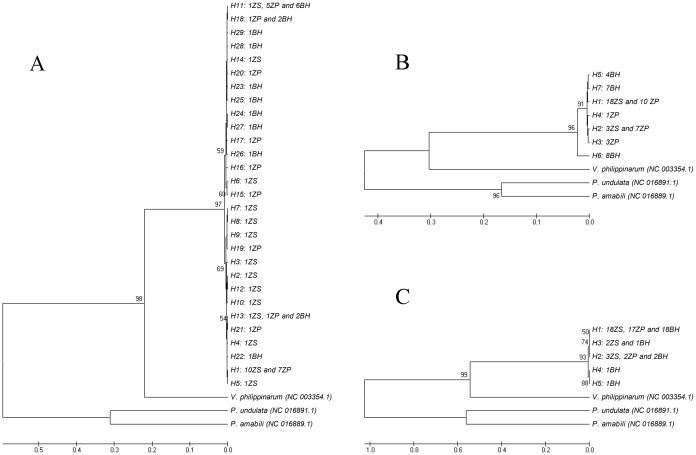
Molecular phylogenetic trees for *M*. *multifarius* by using UPGMA based on COI (A), 12S rRNA (B), and NDI (C) gene sequence data using bootstrap test. Figures before population codes, which are behind the haplotypes, indicate that the number of individuals from the population belongs to the haplotype. All haplotypes are clustered in 1 branch, and the analyzed samples of *M*. *multifarius* are monophyletic with respect to the 3 out-groups: *V*. *philippinarum* (NC 003354.1), *P*. *undulata* (NC 016891.1), and *P*. *amabilis* (NC 016889.1) (100% bootstrap support).

The phylogenetic relationship of *M*. *multifarius* among the haplotypes was determined using COI, 12S rRNA, and ND1 genes of *Venerupis philippinarum* (NC 003354.1), *Paphia undulata* (NC 016891.1), and *Paphia amabilis* (NC 016889.1) as the out-groups (Fig 3). The 3 UPGMA trees showed that most of the haplotypes were weakly associated (less than 50% bootstrap support) or unresolved, which was possibly due to low nucleotide differences among them. Haplotypes of the 3 genes were clustered into 2 obvious branches. Furthermore, the 3 UPGMA trees (COI, 12S rRNA, and ND1 gene) consistently showed the same results: *M*. *multifarius* clustered (bootstrap 98, 96, and 99, respectively) with *V*. *philippinarum* and then clustered with the branch comprising *P*. *undulata* and *P*. *amabilis*.

## Discussion

In this study, significant genetic structure was observed in *M*. *multifarius* populations by using 3 mtDNA markers. This result is consistent with the finding that species with a short planktonic larval stage have a short dispersal distance, especially those with low mobility as adults. Thus, marine bivalves with a short planktonic larval stage could have a strong population genetic structure.

Genetic diversity within and between populations provide a potential genetic resource for future adaptation, and can be critical for the fitness of a population [14]. It is mainly explained by several historical and contemporary processes, such as genetic drift, effective migration, natural selection, fragmentation and range expansion [26]. Significant molecular differentiation in *M*. *multifarius* populations was identified on the basis of all 3 mtDNA markers. Results of the COI gene showed high levels of haplotype diversity (*h =* 0.822–0.889) and low levels of nucleotide diversity (*π* = 0.004–0.006). This pattern is consistent with that observed in previous studies on other species from the East China Sea or South China Sea, for example, *Mytilus coruscus* (*h =* 0.818–0.972 and *π* = 0.00405–0.00747) [27] and the scallop *Chlamys nobilis* (*h =* 0.641–0.966 and *π* = 0.00412–0.01306) [28]. For 12S rRNA (*h =* 0.257–0.678 and *π* = 0.001–0.002) and ND1 (*h =* 0.199–0.391 and *π* = 0.001–0.003) genes, a lower level of genetic variation and fewer haplotypes were obtained (7 haplotypes for 12S rRNA and 5 haplotypes for ND1), largely because they are in a low-variation region of the mitochondrial genome. In our study, the highest level of haplotype diversity was observed in the BH population. This result may be because the bio-resource of BH is more abundant in the southern tropical regions.

Researchers often use *F*_*ST*_ to assess gene flow, and a higher *F*_*ST*_ value indicates a lower level of gene flow (*N*_*m*_) and higher genetic differentiation among populations [29]. *F*_*ST*_ reflects the level of inbreeding within populations [30] or the extent to which populations are differentiated [31]. The presence of genetic structure is an outcome of limited gene flow and a high level of genetic drift within each reproductively isolated group. *F*_*ST*_ values below 0.05 indicate negligible genetic differentiation, whereas values greater than 0.25 indicate high genetic differentiation within the analyzed population [32]. High and significant *F*_*ST*_ values were obtained among different localities of *M*. *multifarius* populations on the basis of both COI (*F*_*ST*_ = 0.100–0.444, *p <* 0.05) and 12S rRNA (*F*_*ST*_ = 0.200–0.742, *p <* 0.05) genes; this, indicated a high degree of genetic differentiation among the *M*. *multifarius* populations. In addition, the highest *F*_*ST*_ values for both COI and 12S rRNA genes were between ZS and BH populations, which endorsed a signal of isolation due to distance. Populations may be divided by major geographic barriers such as land barriers and oceanographic patterns. Our result also showed a higher level of variation when compared with those of other bivalve studies, for instance, Li et al. [27] characterized the genetic relationship of the mussel *M*. *coruscus* in the East China Sea by using the COI gene (*F*_*ST*_ = -0.03612–0.09774), and it showed no significant phylogeographic structure. The planktonic larval stage of *M*. *coruscus* was 35 days [33], and it potentially disperses over larger distances. Similarly, the *F*_*ST*_ value using the COI gene among populations of the mussel *Mytilus galloprovincialis* along the coast of Eastern and Southern China was between -0.0469 and 0.0509 [34]. In contrast, Wang [35] studied the genetic diversity of the clam *Coelomactra antiquata* (planktonic larval stage of only about 9 days) [36] on the basis of the COI gene, and found that the *F*_*ST*_ value between populations from the Bohai Sea and East China Sea was 0.95899, which showed a highly significant genetic differentiation between populations. These results endorsed a highly discussed hypothesis that marine species with an intensive planktonic larval stage would exhibit a high level of genetic differentiation. Nevertheless, with respect to the ND1 data, *F*_*ST*_ values were significant but weak (*F*_*ST*_ < 0.05), and high *N*_*m*_ values were detected among the populations; this may be because the ND1 gene is primitive and high conservative in this species.

Results of the median-joining network suggested an obvious genetic differentiation between ZS and BH populations with respect to the COI and 12S rRNA genes, while results for the ND1 gene showed several ambiguous connections among the 3 populations. Similarly, demographic analyses using UPGMA for all haplotypes show 2 similar branches: one branch contains most (COI) or all (12S rRNA and ND1) haplotypes from the BH population, and the other branch contains most haplotypes from the BH population (COI) or haplotypes from all 3 populations. A much higher degree of genetic differentiation was observed between BH population and the other 2 populations, mostly because BH is located in the Beibu Gulf, South China Sea, and the Leizhou Peninsula (Fig 1) limits the dispersal of planktonic larvae and acts as a barrier to the genetic connectivity of this species. It has been suggested that, in summer, the northward China Coast Current transports propagules from ZP to ZS, although the ZP population is also influenced by the Taiwan Warm Current.

In conclusion, our results showed that differentiations occurred among 3 populations of *M*. *multifarius* on the basis of COI, 12S rRNA, and ND1 gene data; in addition, the BH population was isolated from the other populations. *M*. *multifarius* has a short planktonic larval stage, but it has a high population genetic structure that may facilitate fishery management, captive breeding, and restocking programs for aquaculture and conservation.
